# The Effect of External Treatment of *Arabidopsis thaliana* with Plant-Derived Stilbene Compounds on Plant Resistance to Abiotic Stresses

**DOI:** 10.3390/plants13020184

**Published:** 2024-01-10

**Authors:** Olga A. Aleynova, Zlata V. Ogneva, Andrey R. Suprun, Alexey A. Ananev, Nikolay N. Nityagovsky, Alina A. Beresh, Alexandra S. Dubrovina, Konstantin V. Kiselev

**Affiliations:** 1 Laboratory of Biotechnology, Federal Scientific Center of the East Asia Terrestrial Biodiversity, FEB RAS, 690022 Vladivostok, Russia; aleynova@biosoil.ru (O.A.A.); niknit1996@gmail.com (N.N.N.); a.beresh@mail.ru (A.A.B.); dubrovina@biosoil.ru (A.S.D.); 2 The School of Natural Sciences, Far Eastern Federal University, 690090 Vladivostok, Russia

**Keywords:** auxins, bark, coumaric acid, flavonoids, glycosinolates, piceid, resveratrol, abiotic stress tolerance, Vitis

## Abstract

Stilbenes are a group of plant phenolic secondary metabolites, with *trans*-resveratrol (3,5,4′-trihydroxy-*trans*-stilbene) being recognized as the most prominent and studied member. Stilbenes have a great potential for use in agriculture and medicine, as they have significant activities against plant pathogens and have valuable beneficial effects on human health. In this study, we analyzed the effects of direct application of stilbenes, stilbene precursor, and stilbene-rich extract solutions to the plant foliar surface for increasing the resistance of *Arabidopsis thaliana* to various abiotic stresses (heat, cold, drought, and soil salinity). Exogenous treatment of *A. thaliana* with stilbenes (*trans*-resveratrol, piceid, and spruce bark extract) and phenolic precursor (*p*-coumaric acid or CA) during germination resulted in considerable growth retardation of *A. thaliana* plants: a strong delay in the root and stem length of 1-week-old seedlings (in 1.3–4.5 fold) and rosette diameter of 1-month-old plants (in 1.2–1.8 fold), while the 2-month-old treated plants were not significantly different in size from the control. Plant treatments with stilbenes and CA increased the resistance of *A. thaliana* to heat and, to a lesser extent, to soil salinity (only *t*-resveratrol and spruce extract) to drought (only CA), while cold resistance was not affected. Plant treatments with stilbenes and CA resulted in a significant increase in plant resistance and survival rates under heat, with plants showing 1.5–2.3 times higher survival rates compared to untreated plants. Thus, exogenous stilbenes and a CA are able to improve plant survival under certain abiotic stresses via specific activation of the genes involved in the biosynthesis of auxins, gibberellins, abscisic acid, and some stress-related genes. The present work provides new insights into the application of stilbenes to improve plant stress tolerance.

## 1. Introduction

Stilbenes are natural compounds found in various plant families, such as peanuts, pines, and grapes. These compounds act as plant protection agents and have a structure consisting of two benzene rings and a C6-C2-C6 arrangement [1,2,3]. Many stilbenes are derived from *trans*-resveratrol or *t*-resveratrol, which is a crucial precursor in their production. T-resveratrol can be converted into other stilbenes such as viniferins (through oxidation), pterostilbene (via methylation), or piceid (through glycosylation) [1,4,5,6,7,8]. T-resveratrol also offers several health benefits to humans, including preventing cardiovascular diseases and potentially protecting against certain cancers through antioxidant activity, apoptotic induction, and immune system boosting [9,10,11,12,13,14]. As a result, many research groups have shown great interest in *t*-resveratrol. In grape leaves and berries, *t*-resveratrol acts as a phytoalexin—produced in response to stressors such as pathogen attacks or exposure to ultraviolet (UV) radiation [1].

Several natural sources of stilbenes are known, such as various plants from taxonomically different groups (grapes, spruce, pine, and highlander). This is the main natural source of stilbenes, and the content reaches 23 mg/g of the dry weight (DW) in UV-C treated leaves of *Vitis vinifera* cv. Cabernet Sauvignon [5], 67 mg/g DW in spruce bark *Picea sitchensis* Bong. Car. [15], 84 mg/g DW in core and pine knots of *Pinus sylvestris* L. [16], 250 mg/g DW in bark of spruce *Picea jezoensis* [17].

Cell cultures obtained from the plants described above are also capable of synthesizing stilbenes, but their content is much lower, and high syntheses require inducers or highly stable transgene expression of gene activators of the stilbene biosynthesis. Thus, up to 34 mg/g DW in suspension culture of *Polygonum multiflorum* treated with 100 µM methyl jasmonate (MeJa) [18]; up to 34 mg/g DW in callus culture of grape *Vitis amurensis* Rupr. transformed with the *VaCDPK20* gene [19,20]; up to 38 mg/g DW in suspension and root culture of mulberries *Morus alba* treated with 100 µM salicylic acid (SA) [21].

Alternative sources of stilbenes may be various microorganisms, such as plant endophytes (from grapes) capable of synthesizing stilbenes. Also, laboratory strains of bacteria or yeast into which the genes for stilbene biosynthesis have been transferred. *Escherichia coli* bacteria and *Saccharomyces cerevisiae* yeast with four *Cl* genes from *Nicotiana tabacum* and *STS* from *Vitis vinifera* accumulated up to 16 mg per liter of medium in bacterial cells, up to 6 mg per liter in yeast cells [22]. *Xylaria psidii* fungi, an endophyte from *Vitis vinifera*, accumulated 35 mg per liter of medium [23].

Stilbenes are also found in foods such as wine, table grapes, and peanuts, but their levels are low compared to naturally grown plants. In particular, red wine is known to be the richest source of stilbenes among foods. For instance, Italian red wines contain about 0.6–1.3 mg of stilbenes per liter [24], while French red wines have a concentration of about 0.9–3.8 mg per liter [25]. Russian red wines, on the other hand, exhibit a slightly higher content of stilbenes, ranging from 0.9 to 5.0 mg per liter [26]. Similarly, Brazilian red wines possess stilbenes in a concentration of approximately 0.1–5.4 mg per liter [25].

Stilbenes are also important for plants because plants with high levels of stilbenes are more resistant to adverse conditions: the attack by pathogenic fungi, bacteria, insects, and harmful ultraviolet radiation. However, nothing is known about the effect of stilbenes on plant resistance to other abiotic stresses: cold, heat, drought, and soil salinity. Stilbenes are known to have antioxidant properties, which can positively affect plant resistance to stress. Also, flavonoids, secondary metabolites close to stilbenes, act not only as plant protective substances but also as regulators of plant growth and development, changing the metabolism and transport of auxins [27]. Thus, stilbenes can have a positive effect on plant resistance to abiotic stress, which may be useful in agriculture. Therefore, the aim of this study was to investigate the effect of external stilbenes, phenolic compound precursor, and treatment with stilbene-rich extract water solutions on the growth, development, and resistance of *A. thaliana* plants to abiotic stresses (heat, cold, drought, and soil salinity).

## 2. Results and Discussion

### 2.1. Effect of External Seed Treatment with Stilbenes and p-Coumaric Acid on A. thaliana Growth and Development

**Figure 1:**
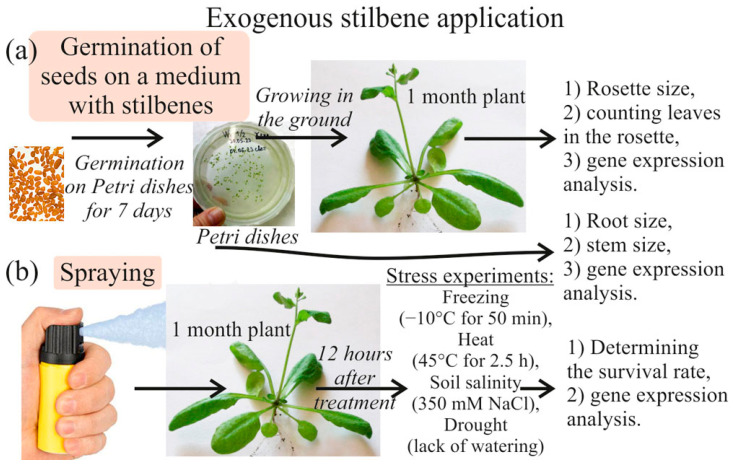
Schematic representation of the treatment of *Arabidopsis thaliana* treatments with exogenous phenolic compounds (*t*-resveratrol, *t*-piceid, *p*-coumaric acid, and bark extract of spruce *Picea jezoensis*). The application of exogenous stilbenes has been utilized as a research tool to investigate its effects on the growth of *Arabidopsis thaliana* seedlings (**a**), as well as to examine changes in the protective properties of *A. thaliana* plants under various abiotic stresses (**b**). The abiotic stresses employed in this study include freezing (−10 °C for 50 min), heat (45 °C for 2.5 h), soil salinity (soaking in 350 mM NaCl), and drought (lack of watering for 2–3 weeks). After applying these treatments, we measured the *A. thaliana* plant rosette, stem, and root sizes, counted the surviving plants, and analyzed the expression levels of genes involved in phytohormone metabolism and stress responses.

The effect of stilbenes and stilbene-containing medications on the growth and development of *A. thaliana* plants was investigated. *A. thaliana* seeds were planted on Petri dishes in the presence of the stilbenes *t*-resveratrol (R) and *t*-piceid (Pi), a stilbene precursor *p*-coumaric acid (CA), and the bark extract of spruce *Picea jezoensis* (BE) (1 and 5 mM, Figure 1a). The dishes were kept in the environmental test chamber under standard temperature and light conditions for 7 days, after which the length of the stems and roots was measured. The seedlings were then planted in separate pots to measure rosette diameter after 1 and 2 months (Figure 1a).

**Figure 2:**
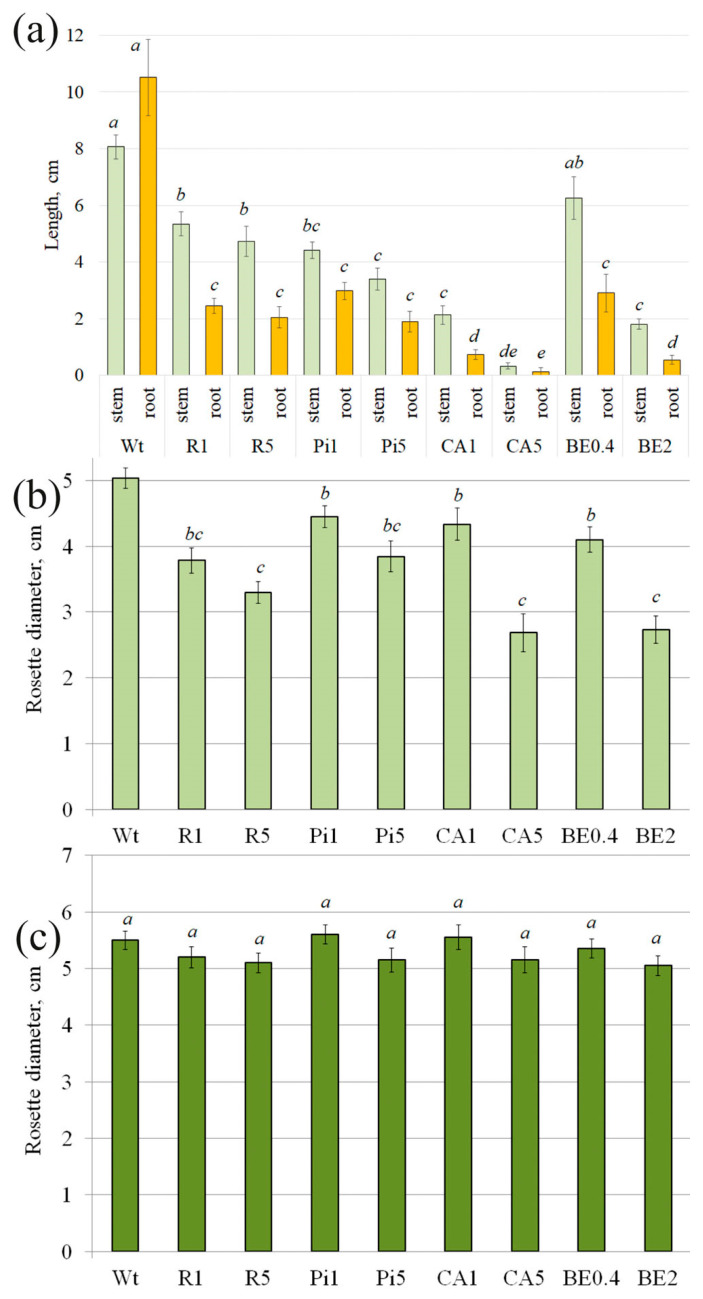
Stem and root length of 7-day-old *Arabidopsis thaliana* seedlings (**a**), grown on a ½ Murashige and Skoog (MS) nutrient medium with the addition of stilbene compounds (*t*-resveratrol or *t*-piceid), or *p*-coumaric acid, or ethanol extract of spruce *Picea jezoensis* bark. The *A. thaliana* seedlings were then transplanted into separate pots and, after 1 (**b**) and 2 months (**c**), were counted rosette diameter. Wt—wild-type *A. thaliana* plants; R1 and R5—*A. thaliana* seedlings or plants, growing on a ½ MS nutrient medium with the addition of *t*-resveratrol 1 and 5 mM, respectively; Pi1 and Pi5—addition of *t*-piceid 1 and 5 mM, respectively; CA1 and CA5—addition of *p*-coumaric acid 1 and 5 mM, respectively; BE0.4 and BE2—addition of extract of spruce bark 0.4 and 2 g/L, respectively. The data are presented as mean ± SE (two independent experiments with twenty technical replicates). Means followed by the same letter were not different using one-way analysis of variance (ANOVA), followed by the Tukey HSD multiple comparison test (*p* < 0.05).

Exogenous treatment of *A. thaliana* seeds with stilbenes (R, Pi, BE) during germination resulted in a strong delay in the root and stem length of 1-week-old *A. thaliana* and rosette diameter of 1-month-old plants—in 1.3–4.5 fold and 1.2–1.8 fold, respectively (Figure 2a,b). The highest inhibition of the growth parameters of the 1-week- and 1-month-old plants was observed at 0.5 mM CA—24.3–79.1 and 1.9 times, respectively (Figure 2a,b). However, the rosette size of the 2-month-old treated plants was not significantly different from that of the water-treated control (Figure 2c).

### 2.2. Influence of External A. thaliana Plant Treatment by Plant-Derived Stilbene Compounds on Survival Rate after Lethal Abiotic Stress Exposure

**Figure 3:**
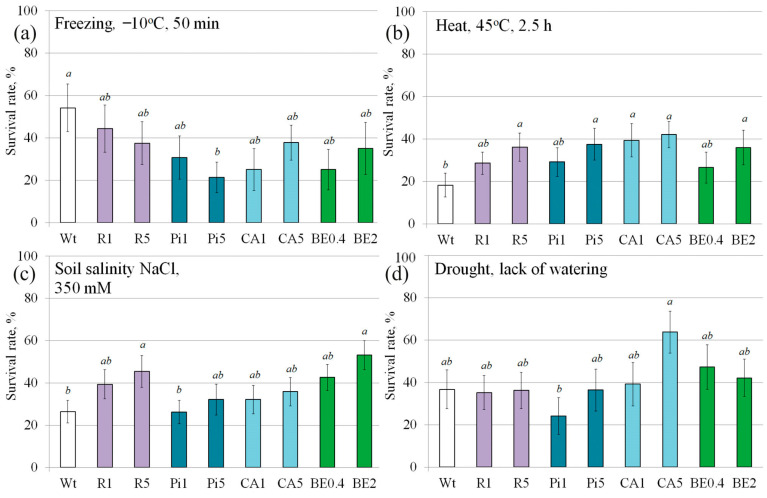
Responses to freezing (**a**), heat (**b**), soil salinity (**c**), and drought (**d**) of wild-type *Arabidopsis thaliana* plants treated with water (Wt), by 1 and 5 mM *t*-resveratrol (R1 and R5, respectively), by 1 and 5 mM *t*-piceid (Pi1 and Pi5, respectively), by 1 and 5 mM *p*-coumaric acid (CA1 and CA5, respectively), by 0.4 and 2 g/L extract of spruce bark (BE0.4 and BE2, respectively). The data are presented as mean ± SE (five independent experiments with twenty technical replicates). Means followed by the same letter were not different using one-way analysis of variance (ANOVA), followed by the Tukey HSD multiple comparison test (*p* < 0.05).

We conducted a study on the main types of abiotic stresses that are common in nature, which often lead to losses in agriculture. The specific conditions for treating these stresses have already been established in a previous study conducted by Dubrovina et al. 2015 [28]. In our experiment, we subjected one-month-old plants to stilbenes and CA treatments for 12 h at a temperature of 22 °C during the night. Following the stress treatment, we observed a decrease in plant survival rates after exposure to cold (Figure 1b). Although all the solutions used resulted in reduced plant survival, the decrease was not statistically significant in most cases. However, when treated with a 5 mM *t*-piceid solution, the plant survival rate decreased by 2.5 times or 60.6% (Figure 3a).

Abiotic stresses are non-living factors, such as temperature extremes or chemical exposure, that can adversely affect plant growth and development. These stresses are a significant concern in agriculture as they can lead to reduced crop yields and economic losses. The study mentioned in the text aimed to explore the effects of different stress treatments on plant survival rates, specifically focusing on the impact of stilbenes and CA.

The results showed that while most treatments did not significantly reduce plant survival, the application of a 5 mM *t*-piceid solution had a substantial negative effect. This research provides valuable insights into the response of plants to abiotic stresses and may contribute to the development of strategies to mitigate their detrimental effects in agriculture.

When exposed to heat, treating plants with stilbenes and CA resulted in a notable increase in plant resistance and survival rates, with plants showing 1.5–2.3 times higher survival rates compared to untreated plants (Figure 3b). This increase was statistically significant, especially when high doses (5 mM) of these substances were used. Similarly, in the case of soil salinization, treatment of *A. thaliana* plants with high concentrations (5 mM) of t-resveratrol and spruce extract had a positive impact on plant survival (Figure 3c). The survival rate of plants treated with these substances increased by 1.7–2.1 times compared to plants treated with water alone. However, under drought conditions, only one sample with a high concentration of CA (5 mM) showed a significant 1.7-fold increase in plant survival compared to the control group treated with water (Figure 3d).

These findings highlight the potential of substance treatment to improve plant survival under different stressors, particularly heat and soil salinization. Further research is needed to investigate the efficacy of these treatments under various conditions and to identify the mechanisms behind their positive effects on plant survival. 

### 2.3. Alteration of Gene Expression of Phytohormone Metabolism and Stress-Associated Genes after Treatment with p-Coumaric Acid and Stilbene Compounds

**Figure 4:**
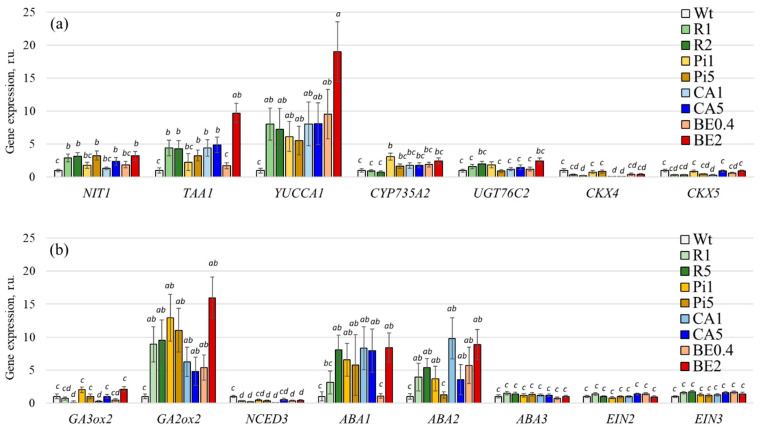
Expression of the phytohormone metabolism genes *AtNIT1*, *AtTAA1*, *AtYUCCA1*, *AtCYP735A2*, *AtUGT76C2*, *AtCKX4*, *5* (**a**), *AtGA3ox2*, *AtGA2ox2*, *AtNCED3*, *AtABA1*, *2*, *3*, *AtEIN2*, *3* (**b**) in *Arabidopsis thaliana* seedlings treated by stilbenes or *p*-coumaric acid. Wt—wild-type *A. thaliana* seedlings; R1 and R5—*A. thaliana* seedlings grown on a ½ Murashige and Skoog (MS) nutrient medium with the addition of *t*-resveratrol 1 and 5 mM, respectively; Pi1 and Pi5—addition of *t*-piceid 1 and 5 mM, respectively; CA1 and CA5—addition of *p*-coumaric acid 1 and 5 mM, respectively; BE0.4 and BE2—addition of extract of spruce bark 0.4 and 2 g/L, respectively. The data are presented as mean ± SE (two independent experiments with twenty technical replicates). Means followed by the same letter were not different using one-way analysis of variance (ANOVA), followed by the Tukey HSD multiple comparison test (*p* < 0.05).

First, we examined the impact of different stilbene solutions on *A. thaliana* plants by analyzing the expression of certain genes involved in phytohormone metabolism. In particular, we focused on genes related to auxin, cytokinin, gibberellin, abscisic acid, and ethylene metabolism (Table S1). Thus, we analyzed three genes involved in auxin metabolism (*AtNIT1*, *AtTAA1*, and *AtYUCCA1*) [29,30], four genes involved in cytokinin metabolism (*AtCYP735A2*, *AtUGT76C2*, *AtCKX4*, and *AtCKX5*) [31,32,33], two genes involved in gibberellin metabolism (*AtGA3ox2* and *AtGA2ox2*) [34,35], four genes involved in abscisic acid metabolism (*AtNCED3*, *AtABA1*, *AtABA2*, and *AtABA3*) [36,37,38], and two genes involved in ethylene metabolism (*AtEIN2* and *AtEIN3*) [39,40] (Figure 4).

We showed that stilbenes and CA significantly increased the expression of a number of the phytohormone biosynthesis genes, such as auxin biosynthesis genes (all three genes analyzed), gibberellin biosynthesis genes (one of two genes analyzed), and abscisic acid biosynthesis genes (two of three genes analyzed) (Figure 4). However, the expression of genes related to gibberellin and ethylene metabolism did not show significant changes (Figure 4).

Interestingly, the effect of stilbenes and CA treatment on the expression of these genes was similar. This suggests that if the treatment had a positive effect on gene expression, most of the compounds used in the experiment had a positive effect as well. This could be attributed to the presence of phenolic rings in the structure of all the treatments used, which may contribute to their beneficial effects on phytohormone biosynthesis.

**Figure 5:**
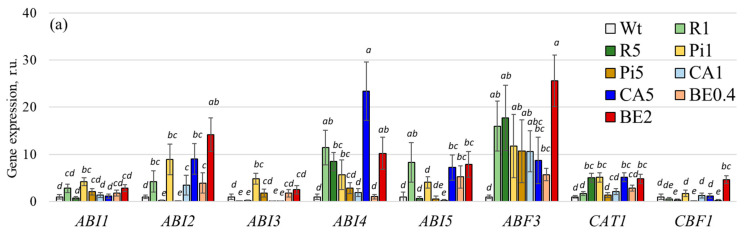

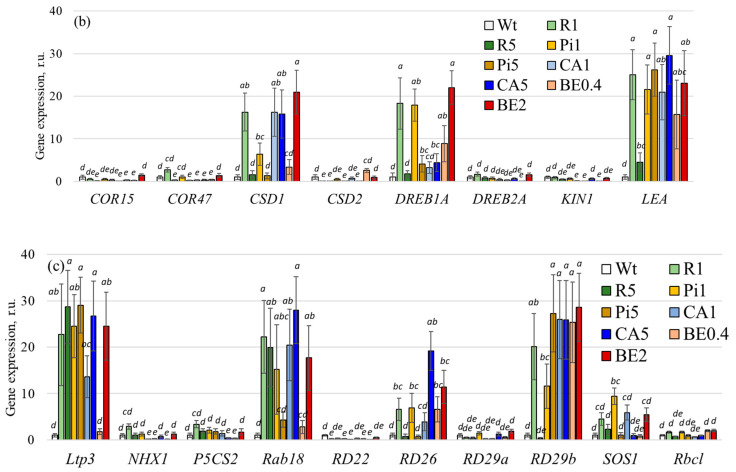
Expression of the stress-responsive genes *AtABI1*, *2*, *3*, *4*, *5*, *AtABF3*, *AtCAT1*, *AtCBF1* (**a**), *AtCOR15*, *47*, *AtCSD1*, *2*, *AtDREB1A*, *2A*, *AtKIN1*, *AtLEA* (**b**), *AtLtp3*, *AtNHX1*, *AtP5CS2*, *AtRab18*, *AtRD22*, *26*, *29a*, *29b*, *AtSOS1*, and *AtRbcl* (**c**) in Arabidopsis thaliana seedlings treated by stilbenes or *p*-coumaric acid. Wt—wild-type A. thaliana seedlings; R1 and R5—*A. thaliana* seedlings grown on a ½ Murashige and Skoog (MS) nutrient medium with the addition of t-resveratrol 1 and 5 mM, respectively; Pi1 and Pi5—addition of *t*-piceid 1 and 5 mM, respectively; CA1 and CA5—addition of *p*-coumaric acid 1 and 5 mM, respectively; BE0.4 and BE2—addition of extract of spruce bark 0.4 and 2 g/L, respectively. The data are presented as mean ± SE (two independent experiments with twenty technical replicates). Means followed by the same letter were not different using one-way analysis of variance (ANOVA), followed by the Tukey HSD multiple comparison test (*p* < 0.05).

Then, in the Arabidopsis plants exposed to stilbenes and CA, we evaluated the expression of the stress-responsive genes (Figure 5; Table S2), encoding transcriptional factors (*AtABI3*, *4*, *5*, *AtCBF1*, *AtDREB1A*, *2A*, *RD26*) [41,42,43,44], protein phosphatases (*AtABI1*, *2*) [41], abscisic-binding factor (*AtABF*) [45], dehydrins (*AtCOR15*, *17*, *AtRab18*, *AtLEA*) [46,47,48], osmolyte synthases (*AtP5CS2*) [49], antioxidant genes (*AtCAT*, *AtCSD1*, *2*) [50,51], protein kinases (*AtKIN1*) [52], lipid transfer proteins (*AtLtp*) [53], ion transporter genes (*AtNHX1*, *AtSOS1*) [54,55]. Also, several genes have no information on the physiological role, but it was shown that their expression strongly increased under stressful influences (*AtRD22*, *29a*, *29b*) [56,57]. In addition, we also showed the expression of the gene of the ribulose-1,5-bisphosphate carboxylase/oxygenase large subunit or RuBisCo, *AtRbcL* gene [58]. This enzyme plays a crucial role in the light-independent phase of photosynthesis, especially in carbon fixation. It helps convert atmospheric carbon dioxide into energy-rich molecules such as glucose. Normally, the expression of *AtRbcL* is consistently high in healthy plants and can influence overall plant development.

Our research revealed that the use of stilbenes and CA significantly increased the expression of about half of the stress-responsive genes we analyzed. This indicates that these treatments have a strong influence on the plant defense system (Figure 5). Specifically, the expression of genes related to abscisic insensitive proteins and abscisic-binding factors (AtABI2, 4, 5, and AtABF3), antioxidant genes (AtCAT1, AtCSD1), transcription factors (AtDREB1A, AtRD26), dehydrin genes (AtLEA, AtRab18), lipid transfer protein gene (AtLtp3), desiccation-responsive genes without known physiological role (AtRD29b), and ion transporter genes (AtSOS1) were significantly increased after treatment with stilbenes and CA (Figure 5).

Interestingly, the effects of the applied substances on the stress-responsive genes were similar to their effects on the phytohormone biosynthesis genes. When a treatment had a positive effect, most of the compounds used also had a positive effect. This can be attributed to the presence of phenolic rings in the structure of these compounds. Phenolic rings are known to have beneficial effects, which explains the positive results observed in our study.

### 2.4. Changes in the Total Phenolic Content of the A. thaliana Plants after Heat, Stilbene, and p-Coumaric Acid Treatment

**Figure 6:**
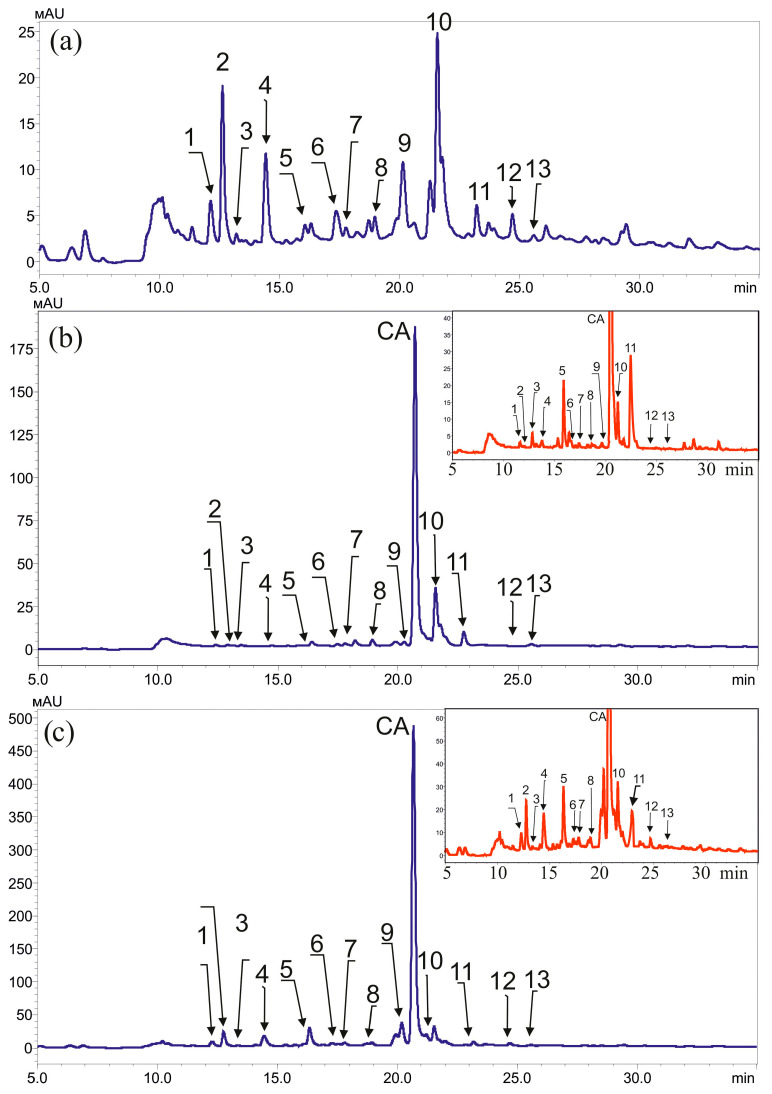
HPLC-UV chromatographic profiles (310 nm) for the methanolic extracts of *Arabidopsis thaliana* plants in normal conditions (**a**) and after treatment with 5 mM *p*-coumaric acid (**b**) and with 5 mM *p*-coumaric acid (CA) and after high temperature with CA (45 °C, 2.5 h) (**c**). Isorhanetin (1, retention time 12.1 min), Indole-3-butyric acid (2, 12.6 min), 7-methysulfinylheptyl glucosinolate (3, 13.2 min), 3-indolylmethyl glucosinolate (4, 14.4 min), glucohirsutin (5, 16.1 min), 4-methoxy-3-indolylmethyl glucosinolate (6, 17.3 min), sinapoyl hexoside (7, 17.3 min), kaempferol hexose dideoxyhexose (8, 19.0 min), kaepferol-3-O-hexoside (9, 20.1 min), kaepferol-3,7-O-diramniside (10, 21.3 min), sinapoyl malate (11, 21.6 min), 1,2-di-o-sinapoyl-beta-o-glucose (12, 24.7 min), sinapic acid (13, 26.1 min).

After exposing the plants to high temperatures (45 °C, 2.5 h), we observed that stilbenes and CA had the most positive effect on enhancing plant resilience. Intrigued by this discovery, we decided to further investigate the phenolic composition of the plants under normal conditions, as well as after exposure to stilbenes, CA, and heat treatments (Figure 6).

Using HPLC-UV chromatographic profiling at 310 nm, we analyzed the methanolic extracts of *A. thaliana* plants under normal conditions and after treatment with 5 mM CA and high temperatures. Our analysis revealed over 40 distinct peaks (Figure 6); however, we were able to accurately determine and calculate the quantities of only 13 of these peaks (Table S3).

Among these 13 substances were substances related to flavonoids (four flavonols: isorhanetin, kaempferol hexose dideoxyhexose, kaepferol-3-O-hexoside, kaepferol-3,7-O-diramniside), plant hormone (auxin: indole-3-butyric acid), four glucosinolates (7-methysulfinylheptyl glucosinolate, 3-indolylmethyl glucosinolate, glucohirsutin, 4-methoxy-3-indolylmethyl glucosinolate), four hydroxycinnamic acids (sinapoyl hexoside, sinapoyl malate, 1,2-di-O-sinapoyl-beta-O-glucose, sinapic acid) (Figure 6, Table S3).

**Figure 7:**
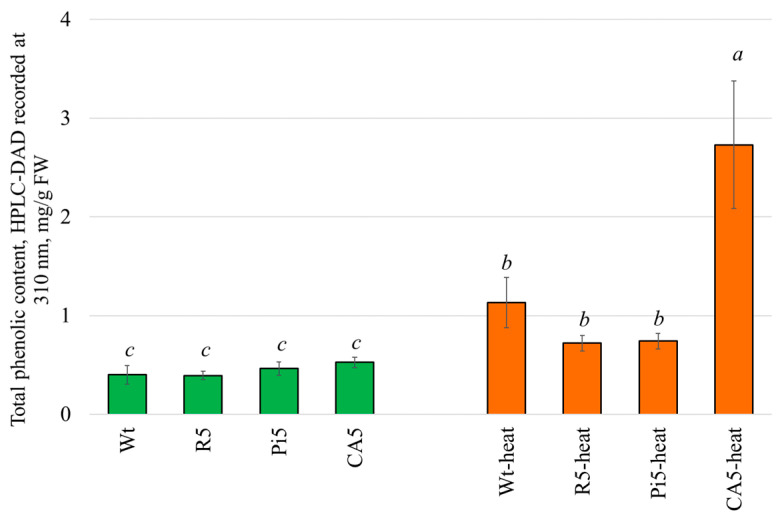
Change in the total content of the determined phenolic compounds in Figure 6 and Table S3 after stilbenes, *p*-coumaric acid (CA), and heat treatment. Wt—total phenolics were extracted 36 h after *A. thaliana* plants treatment with water; R5—by 5 mM *t*-resveratrol; Pi5—by 5 mM *t*-piceid; CA5—by 5 mM CA; Wt-heat—total phenolics were extracted 36 h after *A. thaliana* plants treatment with water and 24 h after treatment with heat stress; R5-heat—by 5 mM *t*-resveratrol and heat; Pi5-heat—by 5 mM *t*-piceid and heat; CA5-heat—by 5 mM *CA* acid and heat. Means followed by the same letter were not different using one-way analysis of variance (ANOVA), followed by the Tukey HSD multiple comparison test (*p* < 0.05).

After 12 h of treatment of *A. thaliana* plants with stilbenes and CA, there was no significant change in the total content of phenolic compounds in plants. However, a notable increase was observed in the levels of certain individual substances (Figure 7). For instance, *t*-resveratrol exhibited a significant 6-fold increase in the content of glucohirsutin (Table 1). Similarly, 5 mM *t*-piceid led to a substantial 5-fold increase in the levels of 3-indolylmethyl glucosinolate (Table 1). CA, on the other hand, demonstrated remarkable effects, causing a 7-fold rise in glucohirsutin content, an 8-fold increase in kaempferol hexose dideoxyhexose, and a 5-fold boost in kaepferol-3,7-O-tyrannicide (Table 1).

During our extensive experiments, we were able to identify a significant improvement in plant growth through the use of high-temperature treatment. The application of heat treatment to *A. thaliana* led to a substantial increase in the presence of all detected flavonoids (increased by 5–7 times), auxins (increased by 8 times), and two out of four glycosinolates (increased by 6–21 times). The greatest increase in the content was observed for 3-indolylmethyl glucosinolate: its content increased 21-fold with heat and 53-fold with the combined action of heat and coumaric acid (Table 1). Glucosinolates, a class of secondary metabolites found primarily in Brassicaceae, are affected by the changing environments and are increased under heat stress [59]. While there is no clear understanding of the mechanism of action of glycosinolates, it is assumed that there are certain signaling molecules that interact with glycosinolates and may be involved in signal transduction [59]. The fact that a transient allocation and redistribution of glucosinolates is observed in response to environmental changes may indicate that glucosinolate-specific function under abiotic stress is still unclear and requires further attention [59].

The content of four derivatives of hydroxycinnamic substances remained unchanged (Table 1). The application of CA to plants further heightened the levels of the aforementioned substances (flavonoids, auxins, some glycosinolates), whereas the treatment by stilbenes (*t*-resveratrol, piceid) led to a decrease in their content (Table 1).

## 3. Conclusions

This study illustrates the ability of exogenous stilbenes and CA to influence plant growth and development by selectively inducing the expression of crucial genes involved in auxin, gibberellin, abscisic acid biosynthesis, and stress response. Consequently, elevated levels of auxins, gibberellins, and abscisic acid induce specific defense mechanisms in plants governed by these phytohormones. Moreover, the plants treated with stilbenes and CA were more resistant to heat, and there was no such resistance to other stresses (cold, drought, soil salinity). Resistance to heat was accompanied by an increase in the expression of stress-response genes and an increase in the content of detected flavonoids, auxins, and glycosinolates.

It is important to note that it was previously shown that external treatment with stilbenes significantly increased the resistance of plants to the damaging effects of ultraviolet radiation [28]. High temperatures and ultraviolet are usually associated with stresses, so perhaps stilbenes activate processes that have a positive effect on plants for both stresses. Also, the effect of the stilbenes and CA on *A. thaliana* plants used was similar. The majority of the compounds used had a positive effect, which can be explained by the presence of phenolic rings in the structure of all the treatments.

In summary, the data obtained revealed that stilbenes could be used to improve plant resistance during the hot summer period when plants are strongly exposed to high temperatures and ultraviolet radiation.

## 4. Materials and Methods

### 4.1. Plant Material

For experiments, we used seeds of *Arabidopsis thaliana* (L.) Heynh. ecotype Columbia-0, which was stored in our laboratory. To sterilize the seeds, we exposed them to chlorine vapors by adding 3 mL of concentrated HCl to 100 mL of bleach (Sayanskhimplast, 7%, Sayansk, Russia). This process took approximately 40–50 min. The sterilized seeds were then germinated in Petri dishes placed in an environmental chamber at a temperature of +22 °C and a light intensity of 120 μmol m^−2^ s^−1^. We used 1/2 Murashige and Skoog (MS) medium [60] with a pH of 5.6, solidified with 0.8% agar, for germination. After 7–8 days of growth on the MS medium, the seedlings were transferred to commercially available soil (“Universalny”, Fasko, Zelenograd, Russia) and placed in an environmental control chamber (Sanyo MLR-352, Panasonic, Kadoma, Japan). The chamber maintained a 16/8 h day/night cycle, with a temperature of +22 °C and a light intensity of 120 μmol m^−2^ s^−1^.

Initially, we investigated the effects of stilbenes on the growth and development of *A. thaliana* plants. We planted *A. thaliana* seeds in Petri dishes containing different concentrations of stilbenes, including t-resveratrol (R, 1 and 5 mM), *t*-piceid (Pi, 1 and 5 mM), *p*-coumaric acid (CA, 1 and 5 mM), and the bark extract of spruce *Picea jezoensis* (BE, 0.4 and 2 g/L extract of spruce bark). Initially, stilbene solutions were dissolved to 1 M in DMSO (Panreac, Barcelona, Spain) and then in sterile water up to the working concentration. After 7 days in the environmental chamber, we measured the length of the stems and roots of the seedlings. Subsequently, the seedlings were transferred to separate pots, and the rosette diameter was measured after 1 and 2 months of growth (Figure 1a).

In another set of experiments, we used 4-week-old *A. thaliana* plants to study the effects of stress treatment. The plants were treated with aqueous solutions of stilbenes, and after an overnight period, they were subjected to various stresses [61]. We used a 2 mL spray bottle to apply the stilbene solution onto the plants. Each pot contained 4–5 plants, and a total of 4 pots were treated with the 2 mL solution. These experiments aimed to understand the impact of stilbenes on the growth, development, and stress response of *A. thaliana* plants.

### 4.2. High-Performance Liquid Chromatography and Mass Spectrometry Analysis

Identification of all compounds was performed using a 1260 Infinity analytical high-performance liquid chromatography (HPLC system) (Agilent Technologies, Santa Clara, CA, USA), coupled to a Bruker HCT ultra PTM Discovery System (Bruker Daltonik GmbH, Bremen, Germany), equipped with an electrospray ionization (ESI) source. Data for all components of extracts were acquired in negative ions mode under the operating conditions as described [62,63]. All determined components of the extracts were identified as described [63] on the base of UV spectra, recorded with a DAD detector, mass spectral data, and chromatographic separation with reference to the values of their respective standards. The contents of each component were determined by using the external standard method using the five-point regression calibration curves built with the reference standards.

Stilbenes levels were analyzed by HPLC with diode array detection (HPLC-DAD) as described [60]. The extracts were separated on Shim-pack GIST C18 column (150 mm, 2.1-nm i.d., 3 µm part size; Shimadzu, Kyoto, Japan) the on HPLC LC-20AD XR analytical system (Shimadzu, Japan), equipped with an SPD-M20A photodiode array detector. The mobile phase consisted of a gradient elution of 0.1% aqueous formic acid (A) and acetonitrile (B) with the following elution profile: 0 to 35 min 0% of B; 35 to 40 min 40% of B; 40 to 50 min 50% of B; 50 to 65 min 100% of B. 1 µL of the sample extract was injected with a constant column temperature maintained at 40 °C and a flow rate maintained at 0.2 mL/min.

### 4.3. RNA Extraction and Real-Time Quantitative PCR

RNA was extracted by the CTAB-based method as described [64]. cDNAs were produced using the MMLV Reverse transcription PCR Kit with oligo(dT)15 (RT-PCR, Evrogen, Moscow, Russia) as described [65].

The mRNA transcript levels of the transgenes were determined by real-time quantitative PCR (qRT-PCR)—the 2^−ΔΔCT^ method [66] with two internal controls, including *AtGAPDH* (NM_111283.4) and *AtEF* (XM_002864638) [65]. The primers designed for qRT-PCRs are shown in Tables S1 and S2.

qRT-PCR reactions were performed in volumes of 20 µL using the real-time PCR kit (Evrogen), as described [65], containing 1x Taq buffer, 2.5 mM MgCl_2_, 0.2 mM of each dNTP, 0.2 µM of each oligonucleotide primer, 1x SybrGreen I Real-time PCR dye, 1 µL cDNAs, and 1 unit of Taq DNA polymerase (Evrogen). Analysis was performed in DTprime 4M1 Thermal Cycler (DNA-technology, Moscow, Russia) programmed for an initial denaturation step of 2 min at 95 °C followed by 50 cycles of 10 s at 95 °C and 25 s at 62 °C.

### 4.4. Statistical Analysis

During the course of our plant experiments, we conducted a total of three independent trials, each comprising 20 plants per treatment. Additionally, our analysis, through HPLC, involved two separate experiments, each of which consisted of three technical replicates. Furthermore, in order to further validate our findings, we utilized three independent experiments for qRT-PCR, conducting a total of 10 technical replicates (five for *AtGAPDH* and five for *AtEF*). The data were presented as mean ± standard error (SE) and analyzed using one-way analysis of variance (ANOVA), followed by the Tukey HSD multiple comparison test (*p* < 0.05).

## Figures and Tables

**Table 1:** The content of individual determined phenolic compounds in mg per g of the dry weight (DW) in the *Arabidopsis thaliana* plants after stilbenes, *p*-coumaric acid (CA), and heat treatment.

#	Name	Wt	R5-C	P5-C	CA-C	Heat-C	Heat-R	Heat-P	Heat-CA
1	Isorhanetin	0 ^c^	0 ^c^	0.01 ^bc^ ± 0.01	0.01 ^bc^ ± 0.01	0.05 ^abc^ ± 0.02	0.02 ^bc^ ± 0.01	0.02 ^bc^ ± 0.01	0.10 ^a^ ± 0.04
2	Indole-3-butyric acid	0.02 ^c^ ± 0.01	0.01 ^c^ ± 0.01	0.01 ^c^ ± 0.01	0.01 ^c^ ± 0.01	0.16 ^ab^ ± 0.05	0.14 ^ab^ ± 0.04	0.10 ^b^ ± 0.02	0.25 ^a^ ± 0.08
3	7-Methysulfinylheptyl glucosinolate	0.08 ^abc^ ± 0.03	0.04 ^bc^ ± 0.02	0.03 ^c^ ± 0.02	0.03 ^c^ ± 0.02	0.04 ^bc^ ± 0.01	0.03 ^c^ ± 0.01	0.04 ^bc^ ± 0.01	0.10 ^a^ ± 0.03
4	3-Indolylmethyl glucosinolate	0.01 ^d^ ± 0.01	0.01 ^d^ ± 0.01	0.05 ^cd^ ± 0.01	0.01 ^d^ ± 0.01	0.21 ^ab^ ± 0.08	0.10 ^bc^ ± 0.02	0.06 ^cd^ ± 0.02	0.53 ^a^ ± 0.21
5	Glucohirsutin	0.01 ^c^ ± 0.01	0.06 ^bc^ ± 0.03	0.04 ^bc^ ± 0.01	0.07 ^b^ ± 0.03	0.06 ^b^ ± 0.02	0.04 ^bc^ ± 0.01	0.02 ^bc^ ± 0.01	0.65 ^a^ ± 0.15
6	4-Methoxy-3-indolylmethyl glucosinolate	0.05 ^c^ ± 0.02	0.02 ^c^ ± 0.01	0.07 ^bc^ ± 0.02	0.03 ^c^ ± 0.02	0.10 ^ab^ ± 0.03	0.09 ^ab^ ± 0.03	0.09 ^ab^ ± 0.03	0.23 ^a^ ± 0.06
7	Sinapoyl hexoside	0.01 ^a^ ± 0.01	0.01 ^a^ ± 0.01	0.01 ^a^ ± 0.01	0.01 ^a^ ± 0.01	0.01 ^a^ ± 0.01	0.01 ^a^ ± 0.01	0.01 ^a^ ± 0.01	0.04 ^a^ ± 0.02
8	Kaempferol hexose dideoxyhexose	0.01 ^c^ ± 0.01	0.02 ^bc^ ± 0.01	0.03 ^bc^ ± 0.01	0.08 ^ab^ ± 0.02	0.07 ^ab^ ± 0.02	0.03 ^bc^ ± 0.02	0.06 ^b^ ± 0.02	0.15 ^a^ ± 0.04
9	Kaepferol-3-O-hexoside	0.03 ^c^ ± 0.01	0.02 ^c^ ± 0.01	0.02 ^c^ ± 0.01	0.03 ^c^ ± 0.01	0.15 ^b^ ± 0.04	0.03 ^c^ ± 0.01	0.03 ^c^ ± 0.01	0.43 ^a^ ± 0.14
10	Kaepferol-3,7-O-diramniside	0.02 ^b^ ± 0.01	0.03 ^b^ ± 0.01	0.02 ^b^ ± 0.01	0.09 ^a^ ± 0.03	0.11 ^a^ ± 0.04	0.04 ^b^ ± 0.02	0.06 ^ab^ ± 0.03	0.08 ^ab^ ± 0.02
11	Sinapoyl malate	0.14 ^a^ ± 0.04	0.16 ^a^ ± 0.09	0.18 ^a^ ± 0.05	0.19 ^a^ ± 0.07	0.18 ^a^ ± 0.03	0.17 ^a^ ± 0.04	0.21 ^a^ ± 0.06	0.17 ^a^ ± 0.03
12	1,2-di-O-Sinapoyl-beta-O-glucose	0.01 ^a^ ± 0.01	0.01 ^a^ ± 0.01	0.04 ^a^ ± 0.01	0.01 ^a^ ± 0.01	0.02 ^a^ ± 0.01	0.01 ^a^ ± 0.01	0.03 ^a^ ± 0.01	0.02 ^a^ ± 0.01
13	Sinapic acid	0.04 ^a^ ± 0.02	0.02 ^a^ ± 0.01	0.01 ^a^ ± 0.01	0.01 ^a^ ± 0.01	0.01 ^a^ ± 0.01	0.01 ^a^ ± 0.01	0.01 ^a^ ± 0.01	0.01 ^a^ ± 0.01

Wt—total phenolics were extracted 36 h after *A. thaliana* plants treatment with water; R5—by 5 mM *t*-resveratrol; Pi5—by 5 mM *t*-piceid; CA5—by 5 mM *p*-coumaric acid (CA); Wt-heat—total phenolics were extracted 36 h after *A. thaliana* plants treatment with water and 24 h after treatment with heat stress; R5-heat—by 5 mM *t*-resveratrol and heat; Pi5-heat—by 5 mM *t*-piceid and heat; CA5-heat—by 5 mM *CA* acid and heat. Means followed by the same letter in each row in the table were not different using one-way analysis of variance (ANOVA), followed by the Tukey HSD multiple comparison test (*p* < 0.05).

## Data Availability

The data presented in this study are available within the article and Supplementary Materials.

## Supplementary Materials

The following are available online at https://www.mdpi.com/article/10.3390/plants13020184/s1, Table S1: Primers for expression analysis of some genes involved in the regulation of phytohormone metabolism by real-time quantitative PCR (qRT-PCR). Table S2: Primers for expression analysis of some stress-responsive genes by real-time quantitative PCR (qRT-PCR). Table S3: The content of individual stilbenes in mg per g of the dry weight (DW) in the *Arabidopsis thaliana* plants.
