# Supplementary material for: The Effect of External Treatment of Arabidopsis thaliana with Plant-Derived Stilbene Compounds on Plant Resistance to Abiotic Stresses
Source: Plants (Basel). 2024 Jan 10;13(2):184. doi: 10.3390/plants13020184 (PMC10818634; doi:10.3390/plants13020184)
Supplement: Supplementary file 1 [file plants-13-00184-s001.zip › plants-2750972-supplementary.pdf]

Table S1. Primers for expression analysis of some genes involved in the regulation of phytohormone metabolism by real-time quantitative PCR (qRT-PCR)

#	Gene (phytohormone)	Function	Primers	GeneBank	References
1	Nitrilase 1, AtNIT1 (auxin)	NIT1-subfamily has been associated with the conversion of indole-3-acetonitrile into the major plant growth hormone, indole-3-acetic acid (IAA)	5'GGC GTT CAT AAC GAA GAA GGG CGT G, 5'TTC CTT CTC TAT GGC TCC CAT TAC C	NM_180680.3	Lehmann et al. 2017
2	Trypyhophan amonotransferase of Arabidopsis 1, AtTAA1 (auxin)	TAA1 – involved in the shade-induced production of indole-3-pyruvate, a precursor to IAA, a biologically active auxin	5'AAC GCT GCG ACG GAG GAT CG, 5'CGT GGA CGG CGG CTT GAC AA	NM_105724.3	Sato et al. 2022
3	Flavin monooxygenases, AtYUCCA1 (auxin)	YUCCA1 – the member of the YUCCA-family, are the main players in auxin biosynthesis	5'TCC GCA TCG CTC CAA GGT TC, 5'GGA AGT ATG GAT CTG CGT TCT CAC C	XP_002869265.2	Sato et al. 2022
4, 5	Cytokinin oxidase 4 and 5, AtCKX4 and AtCKX5 (cytokinins)	CKX4 and CKX5 – catalyzes the irreversible degradation of cytokinins and in many plant species is responsible for the majority of metabolic cytokinin inactivation	CKX4 5'TGG GTG GAT GTT CTG AAG GCG, 5'ACG TTA CTA ATC TGA GGG CCG T; CKX5 5'GAG CCA TTG GCC GTG CTT CA, 5'AAC CAC CAC ACC GTT CCT CCC	NM_001341977.1 NM_106199.5	Mok, Mok, 2001; Werner et al. 2003; Bartrina et al. 2011
6	Cytochrome P450 monooxygenase, AtCYP735A2 (cytokinins)	CYP735A2 – cytochrome P450 monooxygenases (P450s) that catalyze the biosynthesis of trans-Zeatin	5'CTA AAC CCC GTC TCC TCA CC, 5'CTC TTC CCA TAT TGT TTG GAC C	NM_105381.5	Takei et al. 2004
7	Cytokinin N-glucosyltransferase, AtUGT76C2 (cytokinins)	UGT76C2 – encodes a cytokinin N-glucosyltransferase that is involved in cytokinin homeostasis and cytokinin response in planta through cytokinin N-glucosylation	5'CCA TTA CCG TGA TCC ACA CG, 5'CAC GAA ACG GAG ACT CAG CG	NM_120668.4	Wang et al. 2011
8	Gibberellin 3 beta-hydroxylase, AtGA3ox2 (gibberellin)	AtGA3ox2 – encodes a protein with gibberellin 3 beta-hydroxylase activity, step in gibberellin biosynthesis	5'CTG CCG CTC ATC GAC CTC, 5'AGC ATG GCC CAC AAG AGT G	NM_106683.2	Curaba et al. 2004
9	Gibberellin 20-oxidase, AtGA20ox2 (gibberellin)	AtGA20ox2 – encodes gibberellin 20-oxidase. Involved in gibberellin biosynthesis	5'AGA AAC CTT CCA TTG ACA TTC CA, 5'AGA GAT CGA TGA ACG GGA CG	NM_124560.4	Plackett et al. 2012
10	9-cis-epoxycarotenoid dioxygenase, AtNCED3 (abscisic acid)	NCED3 – encodes 9-cis-epoxycarotenoid dioxygenase, a key enzyme in the biosynthesis of abscisic acid (ABA)	5'AGCTAACCCACTTCACGAGC, 5'CCAATTGACGTTCCTGAAC	NM_112304.3	Behnam et al. 2013
11, 12	Zeaxanthin epoxidase, AtABA1 and xanthoxin dehydrogenase, AtABA2 (abscisic acid)	ABA1 and ABA2 are enzymes involved in the biosynthesis of abscisic acid	ABA1 5'GCT ATG AAG GTG ATC TGC TTG TGG, 5'TTC ATA CCA TTT GGA GCA TCA GC; ABA2 5'ATT GAT CAC TGG AGG AGC CAC AG, 5'ATT ACG AAT ATC AGG GCA CGG TG	NM_180954.3, NM_104113	Milborrow 2001
13	Molybdenum cofactor sulfurase, AtABA3 (abscisic acid)	ABA3 - encodes molybdenum cofactor sulfurase. Involved in the conversion of ABA-aldehyde to ABA, the last step of abscisic acid biosynthesis	5'TCACATCATTGGGCGGTTGT, 5'AGATCTTTCCCTTTACTCTC	NM_001332230	Barrero et al. 2006

14	Ethylene-insensitive 2 transmembrane protein, AtEIN2 (ethylene)	EIN2 – transmembrane protein of unknown biochemical activity, involved in ethylene signal transduction	5'GAGAGTCGGCCTGAGCTTTG, 5'GTGGCTCGCTGGAATCTGA	AF141202	Binder, 2020; Alonso et al. 1999
15	Ethylene-insensitive 3 transcription factor, AtEIN3 (ethylene)	EIN3 – a nuclear transcription factor that initiates downstream transcriptional cascades for ethylene responses	5'ACAGTAGCGGCAACAGGTTC, 5'TTGCTGCTTCTGCTGCATTC	NM_112968	Binder et al. 2004; Binder, 2020

Table S2. Primers for expression analysis of some stress-responsive genes by real-time quantitative PCR (qRT-PCR)

#	Gene	Function	Primers	GeneBank	References
1, 2, 3, 4, 5	*Arabidopsis thaliana* abscisic insensitive genes (ABI), AtABI1, AtABI2, AtABI3, AtABI4, AtABI5	Protein phosphatases (ABI1,2) or transcription factors (ABI3,4,5), products of these genes are involved in the transmission of the abscisic acid signal	ABI1 5'AGC TGC TGA TAT AGT CGT CGT TGA TA, 5'GAG GAT CAA ACC GAC CAT CTA ACA; ABI2 5'GTT CTT GTT CTG GCG ACG GAG C, 5'CCA TTA GTG ACT CGA CCA TCA AG; ABI3 5'CAC AGC CAG AGT TCC TTC CTT TAC T, 5'TAG TTG CTG AGG AAC ACA AAC GG; ABI4 5'ACA AGA TTT CTG ACA TCG AGC TCA C, 5'ATC CAT CTC CAA CCA TAT AAC CCG; ABI5 5'AGA GGG ATA GCG AAC GAG TCT AGT C, 5'GTT CGG GTT TGG ATT AGG TTT AGG	NM_118741.3; NM_001345230; NM_113376.4; AF040959.1; NM_001336591	Leung et al. 1997; Brocard-Gifford et al. 2004
6	*A. thaliana* gene of abscisic-binding factor (ABF), AtABF3	AtABF3 is involved in the stress response to drought, oxidative, cold, heat stress through the regulation of genes involved in stress	5'CAA CAT CAG CAA TGG TAA TAG TGG A, 5'CGT CCG AGG CAA GGT AAG TG	NM_119562	Wang et al. 2016
7	*A. thaliana* C-repeat/DRE binding factor 1 (CBF1), AtCBF1	Transcriptional activator AtCBF1 binds to the DRE/CRT regulatory element and induces cold-regulated (*COR*) gene expression increasing plant freezing tolerance	5'GTT TGG GAT GCC GAC TTT GTT, 5'ACC ATC TCC TTC GCC GTC AT	FJ169278	Heidari 2019
8, 9	Dre-binding protein 1A and 2A, AtDREB1A, AtDREB2A	Transcription factor DREB1A and DREB2A regulates gene transcription in conditions of water shortage, high salt concentrations and cold	DREB1A 5'TGC GTT GGC GTT TCA GGA TG, 5'CAA ACT CGG CAT CTC AAA CAT CG; DREB2A 5'CTG GAG AAT GGT GCG GAA GA, 5'CAG ATA GCG AAT CCT GCT GTT GT	NM_118680;	Mizoi et al. 2019
10, 11	Cold-regulated genes (COR), AtCOR15, AtCOR47	COR genes encode dehydrins, high hydrophilicity prevent the loss of water by the cell and stabilize cellular proteins and their expression is induced by cold stress	COR15 5'AAC TCT GCC GCC TTG TTT GC, 5'AGT CGT TGA TCT ACG CCG CTA A; COR47 5'GAA AAG CTT CAC CGA TCC AA, 5'TAC CGG GAT GGT AGT GGA AA	NM_120623.3; NM_101894.4	Ingram et al. 1996; Thomashow et al. 1998
12	Dehydrin gene Rab18, AtRab18	Overexpression of Rab18 dehydrin gene increased the tolerance of arabidopsis transgenic plants to cold stress	5'GCA GTA TGA CGA GTA CGG AAA TCC, 5CCT TGT CCA TCA TCC GAG CTA GA	NM_126038.3	Puhakainen et al. 2004
13	Pyrroline-5-carboxylate synthase gene, AtP5CS2	AtP5CS2 encoding the enzyme in proline (osmolyte) biosynthesis pyrroline-5-carboxylate synthase (P5CS)	5'AGC AGC CTG TAA TGC GAT GG, 5'AAG TGA CGC CTT TGG TTT GC	OX461207	Fabro et al. 2004

14	Gene of late embryogenesis abundant (LEA)-like protein, AtLEA	AtLEA gene encodes dehydrin, overexpression of LEA genes in yeast and rice increased resistance to water deficiency	5'GAG CAT CTT CGT CGG TCT GGA,<br>5'CTC GTG AGG TTG GTC GGT AGT G	NM_127721.4	Mowla et al. 2006; Smith and Graether, 2022
15, 16, 17	Catalase, AtCAT1, Superoxide dismutases, AtCSD1, AtCSD2	Antioxidant genes:<br>CAT1 - catalase 1;<br>CSD1 - cytosolic superoxide dismutases;<br>CSD2 - chloroplastic superoxide dismutases	CAT1 5'AGC GCT TTC GGA GCC TCG TG,<br>5'GGC CTC ACG TTA AGA CGA GTT GC;<br>CSD1 5'GTT GGT AGG GCT GTT GTT GTC,<br>5'TGG ACC TCC TTA TTA CAT CAA;<br>CSD2 5'TTA GTC TGA CCA CTG GAA ACG C,<br>5'GGA TGC TAA ATA AAC CAA AAT GTA	NM_101914.4;<br>LR782542.1;<br>LR782543.1	Yang et al. 2014; Zhou et al. 2022
18	Protein kinase gene, AtKIN1	The KIN1 gene product is a Ser/Thr protein kinase Low temperatures and exogenous abscisic acid induce KIN1 gene expression.	5'CCA ACA AGA ATG CCT TCC AAG C,<br>5'GCT GCC GCA TCC GAT ACA CT	NM_121601.3	Kurkela and Borg-Franck 1992
19	Gene of lipid transfer protein, AtLtp3	Ltp - lipid transfer protein, enhances drought tolerance by remodeling lipid profiles	5'TTT CGC TTT GAG GTT CTT C,<br>5'TGG AAT GCT AAC ACC GC	NM_125323.5	Zhang et al. 2022
20, 21	Ion transporter genes, AtNHX1, AtSOS1,	NHX1 and SOS1 are stress-responsive ion transporter genes (vacuolar and plasma membrane Na+/H+ antiporters, respectively) whose overexpression in transgenic plants enhanced photoprotection capacity under high salinity and drought conditions and increased salt tolerance	NHX1 5'CCG TGC ATT ACT ACT GGA GAC AAT,<br>5'GTA CAA AGC CAC GAC CTC CAA;<br>SOS1 5'TCG TTT CAG CCA AAT CAG AAA GT,<br>5'TTT GCC TTG TGC TGC TTT CC	NM_122597.3;<br>NM_126259.4	Liu et al. 2010; Yue et al. 2012
22	Responsive to desiccation (RD), AtRD22	No information on the physiological roles. AtRD22 - member of the plant-specific BURP domain family involved in *A. thaliana* drought tolerance	5'CAT GAG TCT CCG GGA GGA AGT G,<br>5'CGG CTG GGG TAA AGA AGT TGT C	NM_122472.4	Harshavardhan et al. 2014
23	Responsive to desiccation (RD), AtRD26	RD26 encodes a NAC transcription factor whose transcription is induced in response to drought and high salinity	5'GAT GTG AAG TTA CTG ATG GGT GAA,<br>5'GCG AGC CAA GTC ACA AGG AG	NM_118875.4	Fujita et al. 2004
24, 25	Responsive to desiccation (RD), AtRD29a, AtRD29b,	No information on the physiological roles. Cold, drought, and salt induced both genes	RD29a 5'ATC ACT TGG CTC CAC TGT TGT TC,<br>5'ACA AAA CAC ACA TAA ACA TCC AAA G;<br>RD29b 5'GGA ATC CGA AAA CCC CAT AGT C,<br>5'GGA GTG AAG GAG ACG CAA CAA G	NM_124610.3;<br>NM_124609.4	Msanne et al. 2011
26	Ribulose-1,5-bisphosphate carboxylase/oxygenase large subunit, AtRbcL	Enzyme involved in light-independent part of photosynthesis, including the carbon fixation by which atmospheric carbon dioxide is converted by photosynthetic organisms to energy-rich molecules such as glucose	5'GAA GCA GGG GCT GCG GTA G<br>5'TCT TCT CCT GGA ACG GGC TC	MK525214	Nivison and Stocking, 1983

Table S3. The content of individual stilbenes in mg per g of the dry weight (DW) in the *Arabidopsis thaliana* plants

#	R time	Name	Group of substances
1	12.1	Isorhanetin	Flavonoid (O-methylated flavonol)
2	12.6	Indole-3-butyric acid	A plant hormone in the auxin family
3	13.2	7-Methysulfinylheptyl glucosinolate	Glucosinolate
4	14.4	3-Indolylmethyl glucosinolate	Glucosinolate
5	16.1	Glucohirsutin	Glucosinolic acid and a sulfoxide
6	17.3	4-Methoxy-3-indolylmethyl glucosinolate	Glucosinolate
7	17.8	Sinapoyl hexoside	Glucosyl hydroxycinnamic acid
8	19	Kaempferol hexose dideoxyhexose	Flavonoid (flavonol)
9	20.1	Kaepferol-3-O-hexoside	Flavonoid (flavonol)
10	21.3	Kaepferol-3,7-O-diramniside	Flavonoid (flavonol)
11	21.6	Sinapoyl malate	Sinapic acid ester, hydroxycinnamic acid
12	24.7	1,2-di-O-Sinapoyl-beta-O-glucose	Sinapic acid ester, hydroxycinnamic acid
13	26.1	Sinapic acid	Sinapic acid, hydroxycinnamic acid
